# Krebs von den Lungen-6 as Disease Severity Marker for COVID-19 Patients: Analytical Verification and Quality Assessment of the Tosoh AIA-360 Compared to Lumipulse G600II

**DOI:** 10.3390/ijerph19042176

**Published:** 2022-02-15

**Authors:** Miriana d’Alessandro, Laura Bergantini, Dalila Cavallaro, Sara Gangi, Paolo Cameli, Edoardo Conticini, Siena COVID Unit, Bruno Frediani, Francesco Dotta, Elena Bargagli

**Affiliations:** 1 Respiratory Diseases and Lung Transplantation Unit, Department of Medical and Surgical Sciences & Neurosciences, University of Siena, 53100 Siena, Italy; laurabergantini@gmail.com (L.B.); dalila.cavallaro@student.unisi.it (D.C.); sara.gangi@student.unisi.it (S.G.); paolocameli88@gmail.com (P.C.); bargagli2@unisi.it (E.B.); 2 Rheumatology Unit, Department of Medicine, Surgery and Neurosciences, University of Siena, 53100 Siena, Italy; conticini.edoardo@gmail.com (E.C.); fredianibruno60@gmail.com (B.F.); 3 Diabetes Unit, Department of Medicine, Surgery and Neurosciences, University of Siena and Fondazione Umberto Di Mario ONLUS-Toscana Life Science Park, 53100 Siena, Italy; francesco.dotta@unisi.it

**Keywords:** Krebs von den Lungen-6, COVID-19, analytical validation

## Abstract

Background: Krebs von den Lungen-6 (KL-6) has been proposed as a disease severity marker of COVID-19. All research articles reported the KL-6 assay detected through Fujirebio reagents by Lumipulse G600/G1200 instrument. In the present study, KL-6 assay was analysed through Tosoh AIA-360 and compared with analytical results by Lumipulse G600 in a population of COVID-19 patients. Materials and methods: Sixty-four patients (median age, IQR 67 (58–76) years), all hospitalized for COVID-19 interstitial pneumonia at Siena COVID Unit. KL-6 was measured by two methods, chemiluminescence enzyme immunoassay (CLEIA) and fluorescent enzyme immunoassay (FEIA) method by Lumipulse G600 II and AIA 360 systems, respectively. Results: KL-6 concentrations evaluated by Lumipulse G600II were significantly higher in severe than those in non-severe patients (*p* < 0.0001) as well as evaluating by AIA360 (*p* < 0.0001). Receiver operating curve (ROC) curve analysis showed that KL-6 concentrations, by Lumipuse G600II, distinguished severe from non-severe COVID-19 patients with an area under the curve (AUC) of 99.8% and the best cut-off value was 448 U/mL. AUROC between severe and non-severe COVID-19 patients using T0 KL-6 concentrations by AIA360 was 97.4% and the best cut-off value was 398 U/mL. According to T0 KL-6 concentrations in COVID-19 patients, Bland–Altman difference analysis revealed a mean bias of 78 ± 174.8; while using T1 KL-6 concentrations in COVID-19 patients, Bland–Altman difference analysis revealed a mean bias of 48 ± 126 (95% limits of agreement −199–295) between the Lumipulse G600 II and the AIA360 systems. Conclusions: In conclusion, our study demonstrated that CLEIA and FEIA methods for serum KL-6 detection are comparable and reliable. KL-6 was confirmed as an easily detectable and effective biomarker to identify severe COVID-19 patients.

## 1. Introduction

Krebs von den Lungen-6 (KL-6) is a one of the transmembrane mucins in the lung expressed by injured or regenerating pneumocytes type II [1,2]. In 1992, the diagnostic division of Eidia Co., Ltd. (Tokyo, Japan) performed a wide-ranging pioneer study on KL-6 as a serum biomarker of lung diseases and these findings led to the development of an enzyme-linked immunosorbent assay (ELISA) to determine the absolute amount of KL-6 in clinical samples [3]. In 1999, a chemiluminescent enzyme immunoassay (CLEIA) system became available to detect serum KL-6 levels in ordinary Japanese clinical settings, but not in Western European countries [4]. At the same time, KL-6 was proposed as diagnostic marker for fibrotic interstitial lung disease (ILD) and over the years, it demonstrated its usefulness as a marker for predictive prognosis and response to antifibrotic therapy [5,6,7,8,9,10,11,12,13,14]. To date, more than 400 publications are available about the role of KL-6 in ILD patients, especially on idiopathic pulmonary fibrosis (IPF), as the most common fibrotic ILD. Moreover, KL-6 has been proposed as bioindicator of acute respiratory distress syndrome (ARDS) and infective pneumonia [15,16,17,18,19]. Since outbreak of the severe acute respiratory syndrome coronavirus 2 (SARS-CoV-2) pandemic, KL-6 has also been proposed as a disease severity marker [20,21,22,23]. The pathogenesis of COVID-19 (as the lung disease caused by SARS-CoV-2 is defined) is unclear, though it is postulated that elevated serum concentrations of proinflammatory cytokines and oxidative stress mediators contribute to lung injury, facilitating the onset of an acute respiratory syndrome (similar to ARDS). Host susceptibility and virus-induced direct cytopathic effects against type I and II pneumocytes are suspected to play a crucial role in mediating and perpetuating lung damage. Our research group firstly reported elevated serum concentrations of KL-6 in critical COVID-19 patients and our results were soon confirmed by other researchers [20,22,23]. We further serially evaluated serum KL-6 behaviour in a population of COVID-19 hospitalized patients during follow-up, in order to investigate its potential role in predicting clinical course of disease [21]. All research articles reported the KL-6 assay detected through CLEIAcleia method by Lumipulse G600/G1200 instrument. In the present study, KL-6 assay was analysed by the fluorescent enzyme immunoassay (FEIA) method through Tosoh AIA-360 and compared with analytical results by Lumipulse G600 in a population of COVID-19 patients. 

## 2. Methods

### 2.1. Study Population

Seventy-five patients (median age, IQR 68 (58–77) years), all hospitalized for COVID-19 interstitial pneumonia from August 2021 to December 2021 at Siena COVID Unit, were prospectively and consecutively enrolled. Patients who did not give informed consent to the study or who had a previous diagnosis of interstitial lung diseases, cancer or chronic obstructive pulmonary diseases were excluded. 

Patients were divided into severe and non-severe groups, according to respiratory impairment and clinical management. All patients in the severe group (median age, IQR 71 (62–79) years) underwent intubation and mechanical ventilation in the COVID intensive care unit (ICU), while non-severe patients (not requiring intubation) included hospitalized subjects requiring pharmacological treatments and oxygen supplementation or non-invasive ventilation.

Eleven out of 75 COVID-19 hospitalized patients were excluded because they were affected by concomitant malignancies. Our sixty-four patients underwent serum sampling specifically for KL-6 assessment on hospital admission (t0). 

After hospital discharge (t1) (median IQR, three (2–5) months), twenty-four patients underwent follow-up evaluations including physical examination, lung function tests, diffusing capacity of the lung for carbon monoxide (DLCO), blood gas analysis and high-resolution computed tomography (HRCT) of the chest. CT features (fibrotic interstitial lung abnormalities, ground glass opacities and air-trapping) were evaluated by on-site radiologists experienced in interstitial lung diseases. All patients gave their written informed consent to the study for clinical data collection. The study was approved by our local ethics committee (C.E.A.V.S.E. Markerlung 17431).

### 2.2. KL-6 Assay

Assay of serum KL-6 is based on agglutination of sialylated carbohydrate antigen with KL-6 mAb reagent (Fujirebio Europe, UK and Tosoh Biosciences). Concentrations (expressed in U/mL) were determined by measuring changes in absorbance as described in previous papers [10,22,24]. KL-6 was measured by two methods, CLEIA and FEIA, by Lumipulse G600 II system and AIA 360, respectively. The tests were conducted according to the manufacturer’s protocol.

### 2.3. KL-6: Quality Control of Analytical Determinations and Comparison of Results

Serum concentrations of KL-6 were determined with two automatic biochemical analyzer, Lumipulse G600 II (Fujirebio, Japan) and AIA 360 (Tosoh Biosciences) system that adopted CLEIA and FEIA method, respectively. 

Precision was evaluated according to CLSI EP5-A3 guidelines [25] using two concentrations of reagent control serum assays: L1 (Fujirebio, Europe) (mean 339 (range 272–406) U/mL and L2 (mean 852 (range 682–1022) U/mL. Control serum assays were performed in duplicate, twice a day for 21 consecutive days. To compare the two instruments, we measured 49 serum samples from hospitalized COVID-19 patients and nine of them followed 3 months from hospital discharge. Comparisons were performed according to CLSI EP9-A3 guidelines [26].

### 2.4. Statistical Analysis

The results are reported as means ± SD or as medians and inter quartiles (25th and 75th percentiles) for continuous variables. The Shapiro–Wilk test showed that the data did not have a normal distribution. To evaluate linearity, the coefficient of determination (R2) was determined by multiple linear regression. To evaluate precision, the coefficient of variation was calculated. Bland–Altman analysis was performed for assessing agreement and bias between systems [27].

One-way ANOVA non-parametric test (Kruskal–Wallis test) and Dunn test were therefore performed for multiple comparisons. The Chi-square test was applied to categorical variables. The validity of KL-6 concentrations used to distinguish severe from non-severe patients was assessed by areas under (AUC) receiver operating characteristic (ROC) curve. Sensitivity, specificity, and positive and negative predictive values (PPV and NPV, respectively) were calculated for cut-offs of the different variables. The Youden index (J = max [sensitivity + specificity − 1]) was used to establish the best cut-offs. Statistical analysis and graphic representation of the data were performed by GraphPad Prism 9.3 and XLSTAT 2021 software.

## 3. Results

### 3.1. Study Population

Demographic and immunologic data COVID-19 population at T0, including KL-6 concentrations, are reported in Table 1.

Stratifying patients according to severity, 29 of our patients needed intensive care unit (ICU) and mechanical ventilation. There was a prevalence of males in both groups: 68.9% and 80% in non-severe and severe patients, respectively. Regarding symptoms, 19 out of 35 (54%) non-severe patients and 18 out of 29 (62%) severe patients showed at least two symptoms at onset (fever and dyspnoea). Twenty-five out of 64 patients were without comorbidities. In particular, the severe group included 12 patients with arterial hypertension, four with diabetes and three with heart failure. Two severe patients died during hospital discharge. Thirteen non-severe patients showed arterial hypertension, three dyslipidaemia and four heart failure.

### 3.2. KL-6 Assay and Analytical Validation

**Figure 1:**
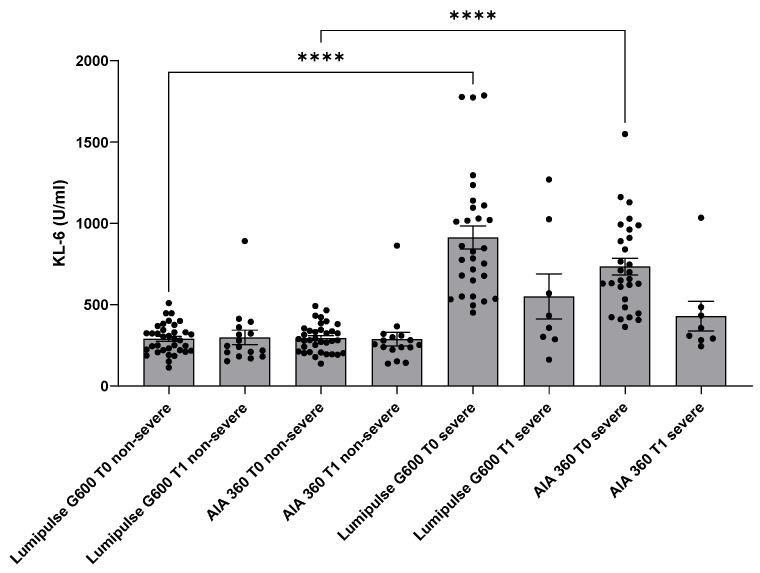
KL-6 concentrations detected by Lumipulse G600II and AIA360 at T0 and T1 stratifying COVID-19 patients according to disease severity. **** *p* < 0.0001.

In the severe group, KL-6 concentrations evaluated by Lumipulse G600II were significantly higher than in non-severe patients (*p* < 0.0001) as well as evaluating through AIA360 (*p* < 0.0001) (Figure 1). 

There were no significant differences of KL-6 concentrations between severe and non-severe patients at T1 evaluating by Lumipulse G600II (395 (291–912) vs. 244 (188–352), respectively) and AIA360 (333 (285–472) vs. 254 (277–308), respectively) (*p* > 0.05).

**Figure 2:**
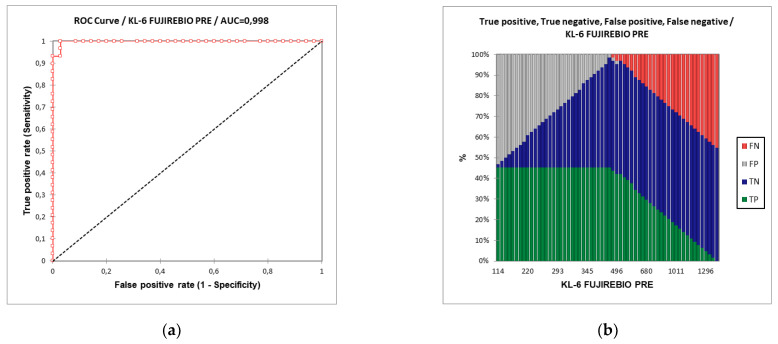
(**a**) Area under the receiver operating curve (ROC) analysis distinguishing severe and non-severe patients according to T0 KL-6 concentrations detected by Lumipulse G600II. (**b**) The percentages of the false negative, true negative, false positive and true positive patients for the KL-6 concentrations at T0 detected by Fujirebio reagent.

Receiver operating curve (ROC) analysis showed that KL-6 concentrations, evaluated by Lumipuse G600II (Figure 2), distinguished severe from non-severe COVID-19 patients with an area under the curve (AUC) of 99.8% and the best cut-off value was 448 U/mL with 97.1% specificity and 100% sensitivity. 

**Figure 3:**
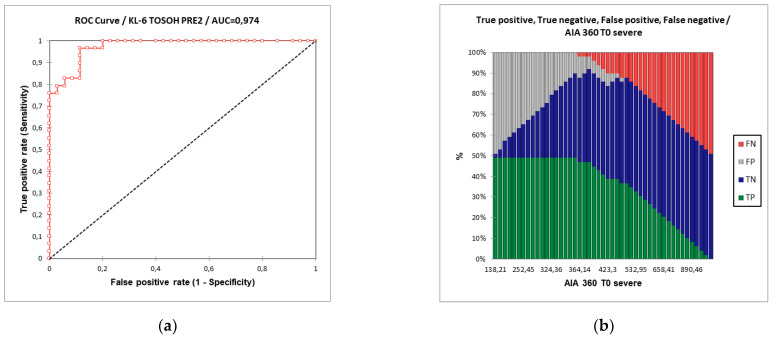
(**a**) Area under the receiver operating curve (ROC) analysis distinguishing severe and non-severe patients according to T0 KL-6 concentrations detected by AIA360. (**b**) The percentages of the false negative, true negative, false positive and true positive patients for the KL-6 concentrations at T0 detected by Tosoh biosciences reagent.

AUROC between severe and non-severe COVID-19 patients using T0 KL-6 concentrations evaluated through AIA360 (Figure 3) was 97.4% and the best cut-off value was 398 U/mL with 89% specificity and 97% sensitivity.

**Figure 4:**
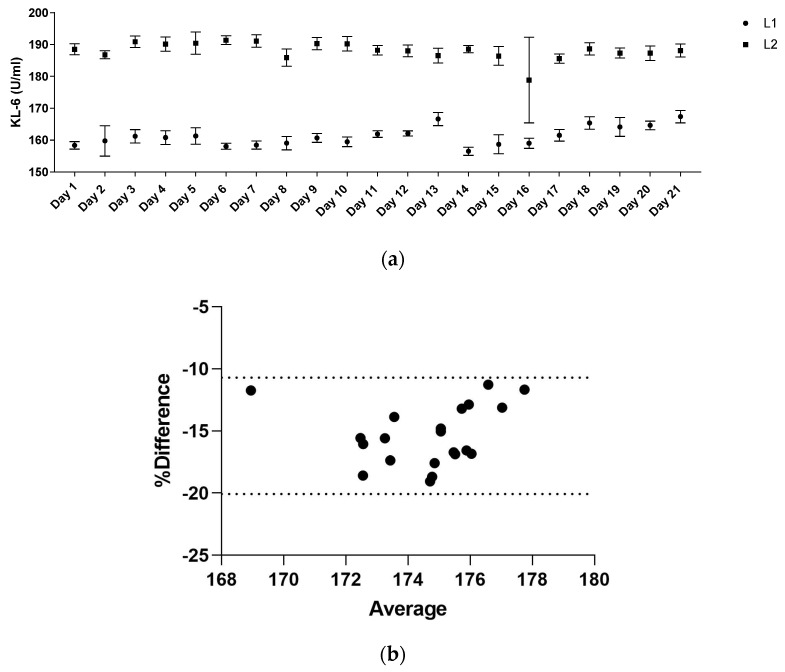
(**a**) the linearity of L1 and L2 reagent concentrations by Fujirebio analysed on AIA360 instrument. (**b**) Bland–Altman analysis for L1 and L2 concentrations for assessing agreement and Bias between systems.

The precision of KL-6 analytical determinations (CV%) for L1 and L2 reagent concentrations (Figure 4a) was 1.84% for the lower and 1.47% for the higher, respectively. Using L1 and L2 reagent concentrations measured by AIA360, Bland–Altman difference analysis (Figure 4b) revealed a mean bias of −15 ± 2.39 (95% limits of agreement −20–−10).

The QQ plot (Figure S1) was obtained using four degrees of freedom (Lumipulse KL-6 T0 and KL-6 T1, AIA360 KL-6 T0 and KL-6 T1) with the coefficient of determination (R2) of the regression analysis of 0.5977. 

**Figure 5:**
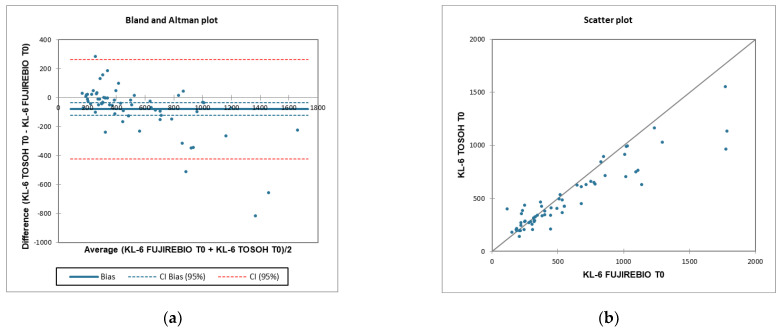
(**a**) Bland–Altman analysis using T0 KL-6 concentrations detected by two methods, CLEIA and FEIA by Lumipulse G600II and AIA360 system, respectively, for assessing agreement and Bias between systems. (**b**) Scatter plot analysis to perform linear correlation between KL-6 T0 Lumipulse G600II and AIA360.

According to T0 KL-6 concentrations in COVID-19 patients, Bland–Altman difference analysis (Figure 5a) revealed a mean bias of 78 ± 174.8 (95% limits of agreement −263.7–421.6) between the Lumipulse G600 II and the AIA360 systems. Figure 5b indicates the regression line between the two systems; correlation coefficient between the two methods is r = −0.661 (95% confidence interval, CI = −0.78, −0.50, *p* = 0.001); that could be evaluated as a very good agreement.

**Figure 6:**
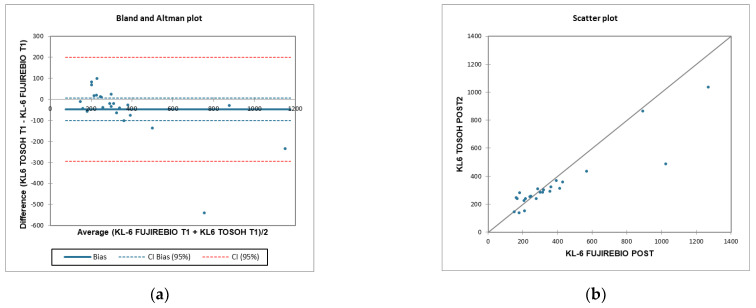
(**a**) Bland–Altman analysis using T1 KL-6 concentrations detected by two methods, CLEIA and FEIA by Lumipulse G600II and AIA360 system, respectively, for assessing agreement and Bias between systems. (**b**) Scatter plot analysis to perform linear correlation between KL-6 T1 Lumipulse G600II and AIA360.

According to T1 KL-6 concentrations in COVID-19 patients, Bland–Altman difference analysis (Figure 6a) revealed a mean bias of 48 ± 126 (95% limits of agreement −199–295) between the Lumipulse G600 II and the AIA360 systems. Figure 6b indicates the regression line between the two systems; correlation coefficient between the two methods is r = −0.642 (95% confidence interval, CI = −0.83, −0.32, *p* = 0.045).

## 4. Discussion

In the present study, the KL-6 concentrations in COVID-19 patients by two different methods, CLEIA and FEIA by Lumipulse G600II (Fujirebio, Europe, Ghent, Belgium) and AIA360 (Tosoh, Biosciences, Tokyo, Japan) system, were evaluated.

Despite the large number of performed studies, no literature is available about the quantification of serum KL-6 through FEIA assay by AIA360. Most articles evaluated KL-6 through CLEIA and enzyme-linked immunosorbent assay (ELISA) assays. In 1992, the diagnostic division of Eidia Co., Ltd. (Tokyo, Japan) performed a wide-ranging pioneer study on KL-6 as a serum biomarker of lung diseases and these findings led to the development of an ELISA that enabled determination of the absolute amount of KL-6 in clinical samples [3]. In 1999, the Japanese Health Insurance Program approved KL-6 as a diagnostic marker of ILD. The CLEIA system became available to detect serum KL-6 levels in ordinary Japanese clinical settings, but not in Western European countries, including Italy. The assay takes only 1 h to perform through the CLEIA method by Lumipulse G600II and the first result is available in 35 min.

FEIA was adopted for the first time in the present preliminary study to evaluate KL-6 concentrations in serum samples from COVID-19 hospitalized patients including a cohort of patients followed after hospital discharge in order to assess the comparison between two methods: CLEIA and FEIA. The usefulness to have another method to perform KL-6 assay in serum samples is extremely important, especially if it takes into account the advantages of the FEIA method through AIA360 instrument in terms of time consumption (36 tests per hour, first result ~ 20 min) and continuous processing.

KL-6 has various pathophysiologic roles (inhibiting cell-cell adhesion of epithelial cells [28] including fibroblast migration [29] and several studies indicated that after bronchial epithelium damage and reparative proliferation processes, KL-6 is released from the regenerated type II pneumocytes into the blood [2,30,31,32,33]. The first analysis of serum KL-6 concentrations was performed by enzyme-linked immunosorbent assay (ELISA) dating to 1992 [3]. Over the years, a CLEIA system [4], that takes only 1 h to be performed, became available to detect serum KL-6 levels in ordinary Japanese clinical settings, but not in Western European countries. 

Increased KL-6 concentrations have been previously reported in different pulmonary diseases, including hypersensitivity pneumonitis [34], idiopathic pulmonary fibrosis [13,14], acute respiratory distress syndrome [15], lung cancer [35], and pulmonary sarcoidosis [8]. Moreover, KL-6 concentrations reflecting the extent of damage and regeneration of type II pneumocytes can predict the risk of illness or death in subjects suffering from lung diseases associated with rheumatologic disorders [11]. A cut-off value of 465 U/mL was recently established by Lumipulse G600II system to distinguish fibrotic ILD patients from healthy subjects and those patients with other non-fibrotic lung diseases. 

Since the outbreak of the SARS-CoV-2 pandemic, our group of researchers suggested for the first time the role of KL-6, detected through the CLEIA method, as a disease severity biomarker for COVID-19 patients and contributed to the definition of the natural course of COVID-19 as the normalization of peripheral KL-6 concentrations during follow-up [21,22]. These data were confirmed in the present study evaluating 64 COVID-19 patients hospitalized between August 2021 to December 2021 at Siena COVID Unit University Hospital by comparing for the first time two different methods, CLEIA and FEIA by Lumipulse G600II and AIA360 system, respectively. The analytical performance of KL-6 assay kit, based on CLEIA and FEIA methods through two different instruments, was confirmed to be reliable: cut-off values discriminating severe from non-severe hospitalized COVID-19 patients were 448 and 398 U/mL for Lumipulse G600 II and AIA360, respectively.

It can be speculated that KL-6 has a crucial role as a key molecule involved in epithelial-mesenchymal interactions, though the pathogenetic pathways involved in serum KL-6 increase are unknown as well as KL-6’s prognostic and predictive role as biomarker in COVID-19 patients.

In conclusion, our study demonstrated that CLEIA and FEIA methods for serum KL-6 detection are comparable and reliable. KL-6 was confirmed an easily detectable and effective biomarker to identify severe COVID-19 patients.

## Figures and Tables

**Table 1:** Demographic and immunological data of COVID-19 hospitalized patients stratified according to disease severity. Abbreviation: WBC, white blood cells; NS, not significant; KL-6, Krebs von den Lungen-6. All data were expressed as median and interquartile range.

Parameters	Severe (*n* = 29)	Non-Severe (*n* = 35)	*p* Value
Age (median *IQR*)	71 (62–79)	63 (57–73)	NS
Gender, M/F	20/9	28/7	NS
T0 KL-6 (U/mL), Lumipulse G600II	827 (599–1103)	293 (220–345)	<0.0001
T0 KL-6 (U/mL), AIA360	658 (508–936)	282 (209–346)	<0.0001
Blood count			
Lymphocytes %	13.6 (6.4–15.9)	18.4 (15.9–22.5)	0.041
Neutrophils %	73.5 (67.5–77.1)	77 (68–81)	NS
Eosinophils %	0.2 (0–1.4)	0.08 (0.02–0.1)	NS
Basophils %	0.15 (0.10–0.17)	0.3 (0.2–0.4)	NS
Monocytes %	7.8 (5.38.5)	7.4 (2.9–7.9)	NS
WBC (cells/µL)	5.2 (3.7–6.9)	4.9 (3.2–7.1)	NS
CRP (mg/L)	5.84 (2.27–6.9)	4.64 (0.64–9.7)	0.037
LDH (U/L)	351 (295–589)	252 (232–330)	NS
Glycemia (mg/dL)	104 (105–224)	96 (76–110)	NS
Lipase (U/L)	24 (14–28)	20 (17–23)	NS
Pancreatic Amylase (U/L)	30 (18–36)	28 (16–42)	NS
AST (U/L)	33 (22–42)	24 (19–39)	NS
ALT (U/L)	32 (13–49)	21 (13–29)	NS

## Data Availability

The data presented in this study are available on request from the corresponding author.

## Supplementary Materials

The following supporting information can be downloaded at: https://www.mdpi.com/article/10.3390/ijerph19042176/s1, Figure S1: The QQ plot obtained using four degrees of freedom (Lumipulse KL-6 T0 and KL-6 T1, AIA360 KL-6 T0 and KL-6 T1) with the coefficient of determination (R2) of the regression analysis of 0.5977.

## Abbreviations

KL-6: Krebs von den Lungen-6; ILD, interstitial lung diseases; COVID-19, coronavirus disease-19; CRP, C-reactive protein.
