# Supplementary material for: Krebs von den Lungen-6 as Disease Severity Marker for COVID-19 Patients: Analytical Verification and Quality Assessment of the Tosoh AIA-360 Compared to Lumipulse G600II
Source: Int J Environ Res Public Health. 2022 Feb 15;19(4):2176. doi: 10.3390/ijerph19042176 (PMC8872393; doi:10.3390/ijerph19042176)
Supplement: Supplementary file 1 [file ijerph-19-02176-s001.zip › ijerph-1550212-supplementary.pdf]

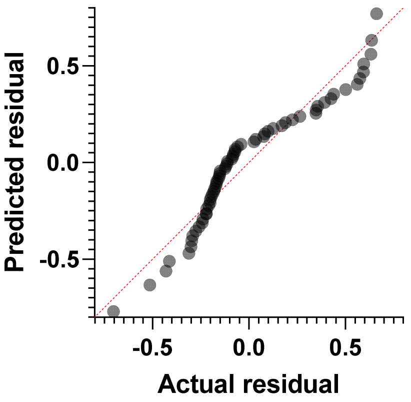

Figure S1. The QQ plot obtained using four degrees of freedom (Lumipulse KL-6 T0 and KL-6 T1, AIA360 KL-6 T0 and KL-6 T1) with the coefficient of determination (R2) of the regression analysis of 0.5977.
